# Improving dog bite victim survey and estimation of annual human deaths due to suspected rabies cases in three selected Liberian cities and environs, 2008–2017

**DOI:** 10.1371/journal.pntd.0008957

**Published:** 2020-12-28

**Authors:** Babasola Oluseyi Olugasa, Nykoi Dormon Jomah, John Bobo Dogba, Olayinka Olabisi Ishola, Ayodeji Oluwadare Olarinmoye, Oluwagbenga Adebayo Adeola, Johnson Funminiyi Ojo, Ali Abdullah Aldosari

**Affiliations:** 1 Centre for Control and Prevention of Zoonoses (CCPZ), University of Ibadan, Ibadan, Oyo State, Nigeria; 2 Department of Veterinary Public Health and Preventive Medicine, Faculty of Veterinary Medicine, University of Ibadan, Ibadan, Nigeria; 3 Central Agricultural Research Institute (CARI), Suakoko, Bong County, Liberia; 4 National Public Health Institute (NPHI), Monrovia, Montserrado County, Liberia; 5 Eng. Abdullah Bugshan Research Chair for Dental and Oral Rehabilitation (DOR), College of Dentistry, King Saud University, Riyadh, Saudi Arabia; 6 Department of Medical Microbiology and Parasitology, College of Medicine and Health Sciences, Bingham University, Karu, *via* Abuja, Nigeria; 7 Department of Statistics, Faculty of Science, University of Ibadan, Ibadan, Oyo State, Nigeria; 8 Department of Geography, College of Arts, King Saud University, Riyadh, Saudi Arabia; Universidad Nacional Mayor de San Marcos, PERU

## Abstract

Rabies remains a public health challenge of unknown magnitude in Liberia in spite of the goal of ensuring that no human in the country dies of rabies by 2030. The annual prevalence of Dog Bite Victims (DBVs) and true load of Annual Human Deaths (AHDs) due to rabies were not known. We investigated three selected cities of Liberia for annual prevalence of DBVs and true load of AHD due to suspected rabies, using 10–year retrospective record, 2008–2017 obtained from Buchanan, Gbarnga, and Voinjama, three socio-economically important cities in post-conflict Liberia. Data were sourced at County Reference Hospitals and at the Liberia National Institute of Health for these cities and their local environs. In addition, household questionnaire survey was used to identify and audit data quality for unreported DBVs, and treatment received from traditional caregivers. The proportion was used to audit the 10-year data on unreported DBVs in the cities. Descriptive statistics was used to summarize annual DBVs over the 10-year period in the three cities, respectively. A standardized clinical decision tree model was used to estimate AHDs due to suspected rabies. Based on questionnaire survey, 140/365, 148/375 and 146/350 DBVs did not visit any orthodox health facility in Buchanan, Gbarnga and Voinjama cities, respectively in 2014. An estimated total of 559 DBVs died of suspected rabies in the three cities and their environs during the 10-year period. Mean yearly prevalence of DBVs was 179±106.82, 393±257.85 and 76.9±38.11 per 100,000 population, while mean AHDs due to suspected rabies was 14.3±8.47, 35.5±23.25, and 6.1±3.21 per 100,000 population in Buchanan, Gbarnga, and Voinjama cities, respectively. The present findings provide annual prevalence of suspected rabies cases, corrected for under-reporting in three selected cities of Liberia. The findings would be useful in planning for stepwise actions towards rabies elimination, ensuring that no human dies of rabies in Liberia by 2030.

## Author summary

Care-seeking preferences of dog bite victims (DBVs) who would rather present to traditional caregivers in rural areas than to conventional hospitals have partly contributed to under-reported cases of suspected rabies in Liberia. For this reason, we reviewed cases of DBVs presented for treatment at human hospitals in Buchanan, Gbarnga and Voinjama cities, and their environs over a 10-year period, 2008–2017. We compared the findings with patronage of traditional caregivers for treatment of dog bite victims among representative households in each of the three cities, based on questionnaire survey and used the ratio derived to correct for under-reporting. Finally, we used a standardized clinical decision tree model to estimate the annual prevalence of human deaths due to suspected rabies cases. We then estimated 559 persons died of suspected rabies in the three cities and their environs during 2008–2017. The average annual prevalence of DBVs was 179±106.82, 393±257.85 and 76.9±38.11, respectively in the three cities, while average annual human deaths due to suspected rabies were 14.3±8.47, 35.5± 23.25, and 6.1±3.21 per 100,000 population, respectively. The mixed-method that we have used in this study for estimating chronologic annual prevalence of human deaths due to suspected rabies is adaptable to other cities of Liberia.

## Introduction

An in-country assessment of Stepwise Action for Rabies Elimination (SARE) protocol was conducted in Liberia in 2018 with a score of 1.5 out of 5 ascribed to the country, indicating that the foundational requirements for developing a national rabies control program has been put in place [1]. Despite this development, the true load of dog bite victims (DBVs) and annual human deaths due to rabies were not known for all, but Monrovia, the capital city of Liberia [2]. The lack of cold facts about the true load of rabies deaths in the country makes the goal of its elimination by 2030 less certain. Efforts to restore public health surveillance in Liberia have been intensified for over a decade commencing five years after the Second Liberian Civil War, which lasted from 1999 to 2003. One Health approach to improving rabies surveillance and control in canine and human populations [3, 4] has gradually improved. Nonetheless, the impact of these efforts might have been small in view of the fact that in Liberia, rabies remains a neglected public health challenge decades after the first report of its clinical diagnosis in DBVs in the country [5].

Based on a recent report of co-circulation of African and Asian lineages of rabies virus in Liberia [6], the epidemiology of rabies in the country might also be undergoing significant changes. The current situation of rabies in Liberia therefore demands more effective One Health action geared towards improving surveillance of DBVs and control of the disease at the human-animal interface, with smart allocation of scarce resources [2, 6, 7]. In order to address the critical aspect of under-reporting of DBVs associated with care-seeking preferences [8, 9], this study was designed to determine the true prevalence of dog bite victims and annual human deaths due to suspected rabies in three selected cities in Liberia, namely Buchanan, Gbarnga and Voinjama.

A One Health collaborative action was endorsed in January 2012, jointly by institutional authorities of Cuttington University, Suakoko, Liberia, the University of Ibadan, Nigeria the Liberian Ministry of Health and Social Welfare, Monrovia, and the Central Agricultural Research Institute (CARI), Suakoko, Liberia [10]. Through this arrangement, some research and technical staff from human and animal disease diagnosis and surveillance units were trained through a graduate program at the Centre for Control and Prevention of Zoonoses (CCPZ), University of Ibadan, and mobilized for improving human-animal disease surveillance in Liberia, with focus on priority neglected zoonoses, especially rabies [11]. The aim of the present study was to improve dog bite victim surveillance in Liberia and develop normalized annual suspected rabies load in the three selected cities for a 10-year period, 2008 to 2017. The specific objectives were to audit retrospective data on dog bite victims, quantify dog bite victims that were presented to orthodox hospitals and those not presented to orthodox hospitals, determine the ratio of both and estimate annual human deaths due to suspected rabies in the three selected cities.

## Materials and methods

### Ethics statement

Ethical approval for spatio-temporal epidemiology of suspected human cases of rabies and habitat suitability for rabies virus circulation was provided by the Liberian Biomedical Research Institute (LIBR) under approval number EC/LIBR/014/039. All data used for analyses (including patient case data) were fully anonymized.

### Study areas

**Fig 1:**
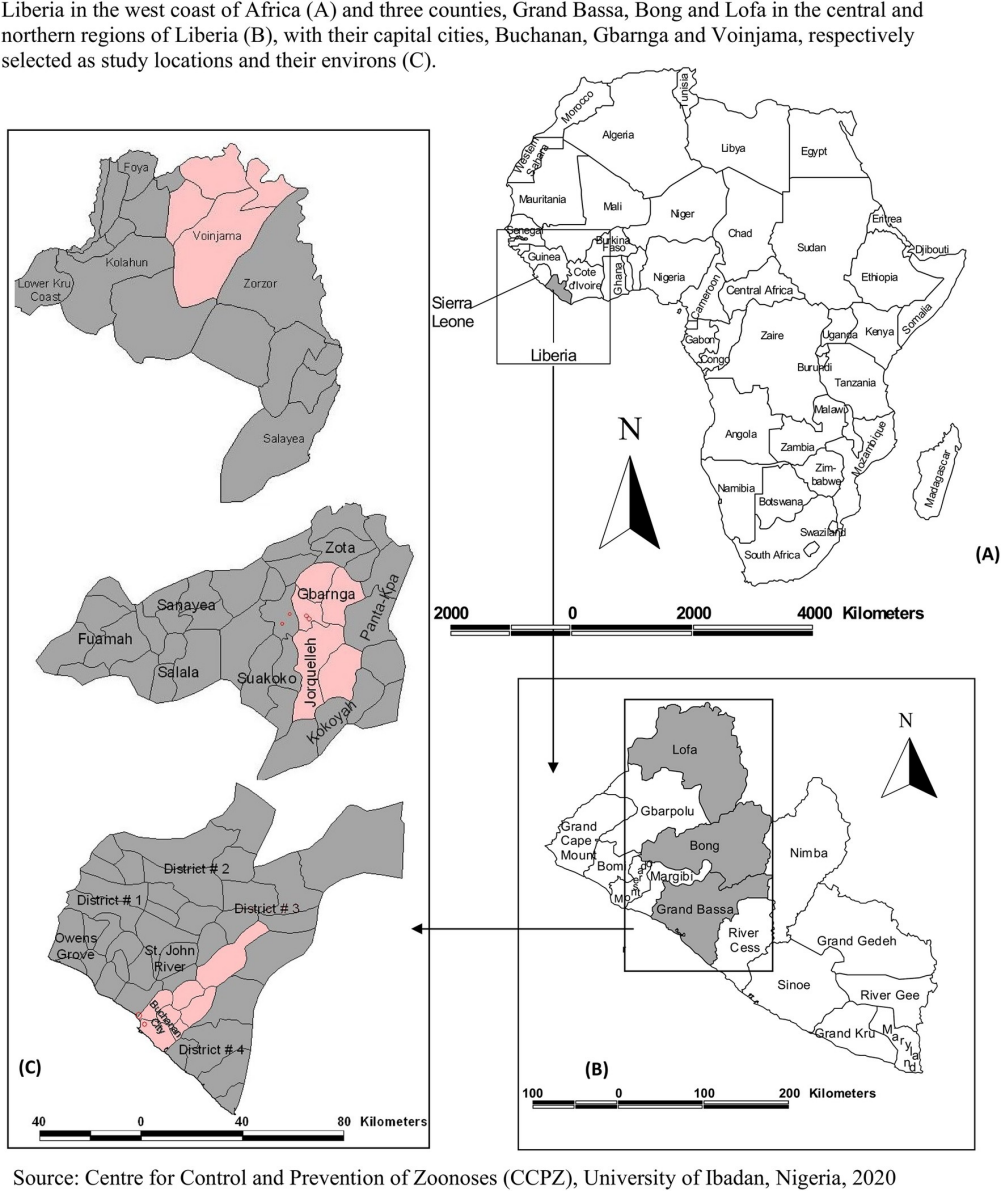
Source: Original map created using ArcGIS 10.1 environment (Environmental Systems Research Institute–ESRI) with supporting datasets from the Liberian Institute of Statistics and Geoinformatics Services.

This study was conducted in three cities selected from three of fifteen counties of Liberia, being three of four counties that submitted DBV reports to the Ministry of Health and Social Welfare during 2008–2013. Findings on the fourth county, Montserrado county, are not included in this study because they have been reported elsewhere [2]. The three cities included in the present study are Buchanan, the capital city of Grand Bassa County; Gbarnga, the capital city of Bong County; and Voinjama, the capital city of Lofa County, Liberia (Fig 1). The first study area, Buchanan and environs (latitude 5.8872° north, longitude 10.0304° west) is a coastal city that shares boundary with the Atlantic Ocean on the southwest, with a population of 34,270 persons in 2008 [12, 13]. Liberia has conducted only four censuses since 1960 till date. They were conducted in 1962, 1974, 1984 and 2008, respectively. Although no other more recent census was conducted in Liberia since 2008, the annual population growth rate in the city from 2009 to 2017 was estimated at 1.4%, 1.5%, and 1.2% in 2008–2011, 2012–2014, and 2015–2017, respectively, in Buchanan and environs. In 2008–2013, reports of cases of DBVs from Buchanan city to the Ministry of Health and Social Welfare (MoHSW) came only from one human hospital, the Liberian Government Hospital (LGH). By 2017, there were nine other clinics in the city that reported DBVs to the Liberian National Institute of Health (LNIH) because of integrated and enhanced disease surveillance and response deployed after Ebola virus disease outbreak in the country [14].

The second city, Gbarnga (latitude 7.0024° north, longitude 9.4728° west) in Bong County, is situated in the central region of Liberia. Gbarnga had a population of 34,046 persons in 2008 [12, 13]. There were no other census figures produced since 2008, but the annual population growth, 2009–2017 for the city was estimated at 1.0% in Gbarnga and environs. In 2008 to 2013, Phebe Hospital and Nursing School (PHNS) alone reported DBV cases to the MoHSW. By 2017, DBVs were reported by 22 other clinics/hospitals, in addition to PHNS [14]. The third study area, Voinjama city and environs (latitude 8.4202^o^ N, longitude 9.7539^o^ W), had an estimated human population of 26,594. The annual population growth estimate for the city, 2009–2017 was 1.3% [12, 13]. The main occupation of its residents was farming. A government health facility, the Tellowayan Memorial Hospital (TMH) alone reported DBVs to the MoHSW in 2008–2013. However, by 2017 some 20 other clinics/hospitals reported DBVs to the LNIH, in addition to TMH [14].

### Study design

The study was based on an explanatory sequential mixed-method design, which included retrospective clinical case review of dog bite victims, household questionnaire survey of DBVs, their care-seeking preferences, and multi-criteria analysis of PEP compliance for estimation of annual human deaths due to suspected rabies cases in three selected cities of Liberia. The three cities were purposively selected because they were among the only four cities that submitted monthly reports on DBVs to the Ministry of Health and Social Welfare (MOHSW) in Liberia from 2008 to 2013. The submission of reports was also partly because they were designated reference hospitals in their respective county. All data were anonymized to protect the privacy of patients. In most cases, residential addresses of DBVs were available but anonymised. The addresses indicated site names that were geospatially referenced to produce map points for a Geospatial Observation-Linked Data on Suspected Rabies Exposure at the Human-Dog interface (GOLD-SREHD) profile.

### Case definition and classification of rabies exposure

During this study, hospital records of DBVs were classified, based on WHO guidelines for case classification of human exposure to rabies, as no exposure, suspected (or possible) exposure, probable exposure, and confirmed exposure cases [15, 16]. Cases of *no exposure* included records of humans touching or feeding animals or having animal licks on intact skin. A case of *suspected exposure* was defined as a person who has had close contact (usually a bite or scratch) with a rabies-susceptible animal in (or originating from) a rabies-infected area. A case of *probable exposure* was defined as a person who has had close contact (usually a bite or scratch) with an animal displaying clinical signs consistent with rabies at the time of exposure, or within 10 days following exposure in a rabies-infected area [17]. A case of *confirmed exposure* is a person who has had close contact (usually a bite or scratch) with a laboratory-confirmed rabid animal [15–17].

### Data sources

#### Review of retrospective hospital case records

The initial dataset comprised all clinical cases of DBVs (n = 618; 460 in Buchanan, 85 in Gbarnga, and 73 in Voinjama) that were presented for treatment at the three selected reference hospitals during the first 6-year period, between January 1, 2008 and December 31, 2013, in the three cities (Table 1). The 2014–2016 DBVs datasets were not collected in Liberia due to Ebola virus disease (EVD) outbreak during that period. Non-dog bite cases and case records of dog bite incidents that occurred outside each of the cities and their immediate environs, not exceeding the county boundaries were excluded from the final dataset used for this study. However, we utilized predicted cases of DBVs from deterministic model of projected data for 2014–2017 dog bite cases. Details about the deterministic model procedure is presented below.

**Table 1:** Age and gender based distribution of dog bite victims presented for post-exposure treatment at selected county-specific reference hospitals reporting for Buchanan, Gbarnga, and Voinjama cities, respectively, Liberia, 2008–2013.

Study area	Age group (years)	Male (%)	Female (%)	Total (%)
Buchanan, Grand Bassa County (n = 460, Liberian Government Hospital)	< 15	119 (56.4)	92 (43.6)	211 (100)
	15–30	71 (63.4)	41 (36.6)	112 (100)
	31–45	33 (47.8)	36 (52.2)	69 (100)
	> 45	34 (54.0)	29 (46.0)	63 (100)
	Missing	1 (20.0)	4 (80.0)	5 (100)
	Total	258 (56.1)	202 (43.9)	460 (100)
Gbarnga, Bong County (n = 85, Phebe Hospital)	< 15	19 (79.2)	5 (20.8)	24 (100)
	15–30	18 (75.0)	6 (25.0)	24 (100)
	31–45	15 (65.2)	8 (34.8)	23 (100)
	> 45	11 (78.6)	3 (21.4)	14 (100)
	Total	63 (74.1)	22 (25.9)	85 (100)
Voinjama, Lofa County (n = 73, Tellewoyon Hospital)	< 15	25 (62.5)	15 (37.5)	40 (100)
	15–30	11 (68.8)	5 (31.3)	16 (100)
	31–45	4 (40.0)	6 (60.0)	10 (100)
	> 45	2 (28.6)	5 (71.4)	7 (100)
	Total	42 (57.5)	31 (42.5)	73 (100)

NB: *Data provided in this table represent a fraction of dog bite victims in each of the three cities*, *being individuals who accessed orthodox hospital care by presenting victims for treatment at County-specific reference hospital*. *The comprehensive normalized dog bite victims corrected for under-reporting in Buchanan*, *Gbarnga and Voinjama cities*, *are provided in Tables* 6, 7 *and* 8, *respectively*.

For each DBV, information on the date of bite incident, gender, age, residential location, injured body part(s), the length of time that elapsed before victim showed up for anti-rabies prophylaxis, medications given, and treatment outcome were extracted from the records. The data generated were sorted on monthly basis for the 10-year period, from January 2008 to December 2017 and used to assess treatment compliance and time of presentation of DBVs for treatment, especially when the bite was assumed to have come from a rabid dog.

#### Household questionnaire survey on DBVs and care-seeking preference

A pre-tested questionnaire on care-seeking preferences of DBVs and their PEP compliance was used for household survey in the three cities, between January and August 2014. A total of 1,090 households (365, 375 and 350, respectively, in Buchanan, Gbarnga and Voinjama) were administered the questionnaire, just at the onset of the West African Ebola virus epidemic in Liberia. A respondent was provided by each household. Respondents were selected based on a three-stage population-representative stratified random sampling method. In each city, human dwellings (that is, stand-alone buildings) were randomly selected based on geographic size (that is, probability proportional to size). Dwellings were considered to contain multiple households if meals or living spaces were not shared. Hence, number of households in each dwelling was determined, and in-house identification number was assigned to each household. A simple random sampling method was then used to select a total of 1090 households enlisted and used for the study. These however were not integrated into the site names that were geospatially referenced to develop the Geospatial Observation-Linked Data Profile of Human Exposure to Suspected Rabid Dogs (GOLD-PHESRD).

#### Deterministic model for forecasting 2014–2016 missed DBV data

Due to the prevailing challenges of EVD epidemic in West African that resulted in severe humanitarian crisis of international concern [14, 18], no human hospital records of dog bite victims in 2014–2016 were available to the authors at the county reference hospitals and at the Liberian NIH. Thus, we utilized predicted case of DBVs from deterministic model of projected data for 2014–2017 expected for Buchanan, Gbarnga and Voinjama. The data were previously developed and reported by co-authors of the present study [19]. A full description of this deterministic model is given in the full text of the paper online (https://www.researchgate.net/publication/302575115). At the resumption of reporting of DBV cases in January 2017, the Liberia Ministry of Health and Social Welfare provided dog bite data for 2017, as collated by the Liberian National Institute of Health (LNIH) which was established in 2016 after the EVD epidemic [14]. We then compared the model projected data for 2017 [19] with the actual cases reported to LNIH in 2017. The findings were evaluated and integrated into a continuous 10-year data-profile of DBVs in the three cities with their respective county environs. The LNIH-enhanced reported data profile for 2017 DBVs comprised hospital-records of DBVs from 10 clinics/hospitals in Buchanan, including LGH; some 23 clinics/hospitals in Gbarnga, including PHNS; and 21 clinics/hospitals in Voinjama, including TMH.

#### One health data audit and rabies laboratory confirmation

Evidence of confirmation of rabies infection among DBVs was sourced from the Liberian National Diagnostic Unit. However, this was limited to identification of clinical cases that were consistent with WHO case definition of rabies. The evidence included neurological signs, hydrophobia in dogs, and/or human death with history of dog bite. By 2017, the Central Veterinary Laboratory installed a fluorescent microscope in collaboration with Swiss Tropical and Public Health Institute and funding by the Global Alliance for Vaccines and Immunizations (GAVI). However, routine confirmatory diagnosis of rabies cases were not performed at that time as both the reagents and manpower for use of the equipment were just being deployed in that year [20, 21].

A field survey and laboratory diagnosis of rabies from suspected human and dog specimens in Liberia, 2014–2016 had utilized RNA extraction technique, PCR detection and gene sequencing protocol [6] to confirm rabies virus infection in dogs and human exposures. The present authors have reported in an earlier paper [6], three rabies virus (RABV) strains isolated during the 2014–2016 survey in Liberia. The three RABVs sequences were accessioned in NCBI GenBank (MF765758, MH507336 and MH507337). However, the great majority of offending dogs and their bite victims were not tested. They remained rabies suspect as recommended by WHO classification for Liberia, being a recognized rabies endemic West African state [15]. Likewise, humans bitten by suspected rabid dogs were rabies exposure suspects

### Conversion of site name to map point of DBVs presented to hospitals

The residential addresses of DBVs were available in most cases and were used to generate map points of street locations of DBVs. A combination of hand-held Global Positioning System (GPS, Garmin eTrex) and Google Earth Pro (Google, California, USA) were used to convert site names of DBVs to map points for all 618 victims enlisted in the study. Table 1 shows the distribution and proportion of 618 DBVs in the cities, including LGH, Buchanan, PHNS, Gbarnga, and TMH, Voinjama having 460, 85, and 73 DBVs, respectively, over the first 6-year period. The map points were saved in Microsoft Excel, version 2007 (Microsoft, Redmond, Washington), and then used for map visualization and spatio-temporal analyses, using Kulldorff’s space-time scan statistics as previously described [22–27].

### Statistical analyses and modelling

#### Descriptive and categorical analysis of DBVs

**Fig 2:**
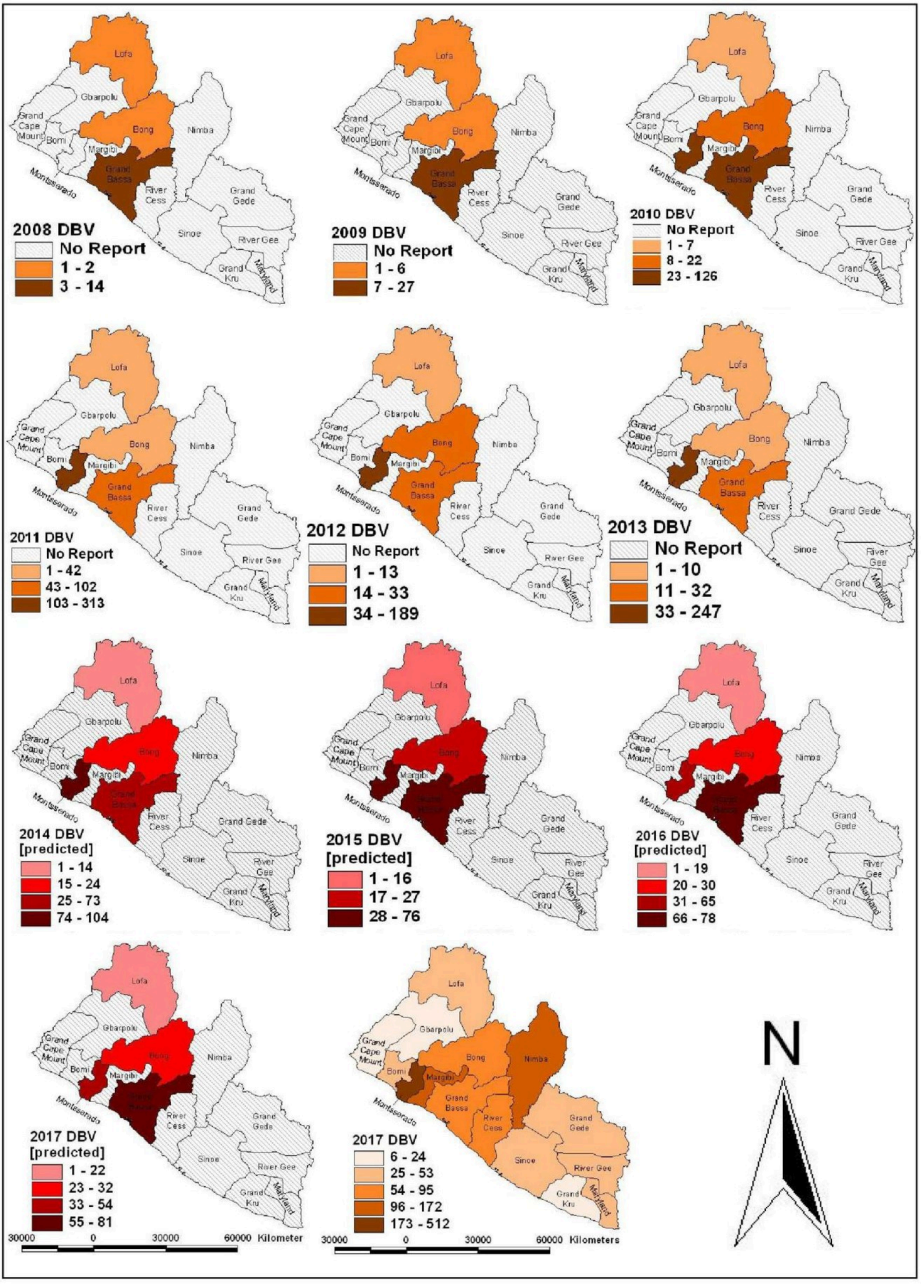
Source: Original map created using ArcGIS 10.1 environment (Environmental Systems Research Institute–ESRI) with supporting datasets from the Liberian Institute of Statistics and Geoinformatics Services.

A descriptive map (Fig 2) of retrospective annual DBVs that were presented for treatment in Liberian cities and environs was drawn to visualize the geographic distribution of reported cases by county health authorities during the 10-year period, 2008–2017, using ArcGIS 10.1 (Environmental Systems Research Institute, ESRI, Redlands, CA) platform. Epidemiological profile of the victims was then compiled to summarize their ages, gender, time of care after bite (categorized into early, when less than 24 hours; or late, when more than 24 hours), site of dog-bite mark on the human body, initial treatment received, number of doses of rabies vaccine and/or number of doses of Equine Rabies immunoglobulin administered. The compliance of DBV to PEP treatment appointments was aggregated into: (i) completed; or (ii) defaulted. In addition, a descriptive statistics of treatment outcomes was computed under three categories, namely: (i) prevented; (ii) dead; and (iii) unknown (Tables 2–4). A multivariate matrix was computed to display the frequency distribution of age, gender, and seasonal frequency of dog bite cases in the three cities. Chi Square test was used to analyze the frequencies of each of the categorical variables. Statistical significance was determined at α_0.05_. DBVs were categorized based on site of bite mark, as bite to head, hands, legs, trunk (Table 5), and multiple sites.

**Table 2:** Profile of dog bite victims and suspected rabies exposed individuals presented for post-exposure treatment at the Liberian Government Hospital, Buchanan City, Liberia, 2008–2013*.

Characteristics	Categories	^+^DBVs (%)	^+^Defaulted in PEP (%)	^+^Completed PEP (%)
Age groups	< 15	154 (46.7)	126 (38.2)	28 (8.5)
	15–30	81 (24.5)	68 (20.6)	13 (3.9)
	31–45	48 (14.5)	41 (12.4)	7 (2.1)
	> 45	47 (14.3)	41 (12.4)	6 (1.8)
Gender	Male	179 (54.2)	143 (43.3)	36 (10.9)
	Female	151 (45.8)	133 (40.3)	18 (5.5)
Time care sought	< 24 hours	101 (30.6)	82 (24.8)	19 (5.8)
	> 24 hours	156 (47.3)	150 (39.4)	26 (7.9)
	Unknown	73 (22.1)	64 (19.4)	9 (2.7)
Anatomical site of bite	Head	69 (20.9)	57 (17.3)	12 (3.6)
	Hands	49 (14.9)	40 (12.1)	9 (2.9)
	Legs	122 (37.0)	107 (32.4)	15 (4.5)
	Trunk	20 (6.0)	18 (5.5)	2 (0.6)
	Multiple sites	70 (21.2)	54 (16.4)	16 (4.8)
Rabies exposure	Not exposed	6 (1.8)	6 (1.8)`	0 (0.0)
	Suspected	259 (78.5)	259 (78.5)	0 (0.0)
	Probable	65 (19.7)	11 (3.3)	54 (15.4)
	Confirmed	0 (0.0)	0 (0.0)	0 (0.0)
Treatment outcome	Prevented	55 (16.7)	1 (0.3)	54 (15.4)
	Death	4 (1.2)	4 (1.2)	0 (0.0)
	Unknown	271 (82.1)	271 (82.1)	0 (0.0)
Observation of dog	Observed	11 (3.3)	6 (1.8)	5 (1.5)
	Not observed (< 1 day)	319 (96.7)	270 (81.8)	49 (14.8)

^+^N = 330 out of 460

*PEP*, Post-exposure prophylaxis

*DBVs*, Dog-bite victims

* Data provided in this table represent a fraction of dog bite victims in Buchanan city and environs, being those that accessed orthodox hospital care by presenting victims for treatment at County-specific reference hospital were enrolled. Normalized dog bite victims for a decade in Buchanan city and environs is provided in Table 6.

**Table 3:** Profile of dog bite victims and suspected rabies exposure patterns presented for post-exposure treatment at Phebe Hospital and Nursing School, Suakoko along Gbarnga city, Liberia 2008–2013*.

Characteristics	Categories	^+^DBVs (%)	^+^Defaulted in PEP (%)	^+^Completed PEP (%)
Age groups	< 15	24 (28.2)	20 (23.5)	4 (4.7)
	15–30	24 (28.2)	23 (27.1)	1 (1.2)
	31–45	23(27.1)	19 (22.4)	4 (4.7)
	> 45	14 (16.5)	13 (15.3)	1 (1.2)
Gender	Male	63 (74.1)	55 (64.7)	8 (9.4)
	Female	22 (25.9)	21 (24.7)	1 (1.2)
Time care sought	<24 hours	12 (14.1)	11 (12.9)	1 (1.2)
	>24 hours	50 (58.8)	47 (55.3)	3 (3.5)
	Unknown	23 (27.1)	18 (21.2)	5 (5.9)
Anatomical site of bite	Head	19 (22.4)	17 (20.0)	2 (2.4)
	Hands	11 (12.9)	10 (11.8)	1 (1.2)
	Legs	30 (35.3)	28 (32.9)	2 (2.4)
	Trunk	11 (12.9)	10 (11.8)	1 (1.2)
	Multiple sites	14 (16.5)	12 (14.1)	2 (2.5)
Rabies exposure	Not exposed	1 (1.2)	1 (1.2)`	0 (0.0)
	Suspected	69 (81.2)	69 (81.2)	0 (0.0)
	Probable	15 (17.6)	6 (7.1)	9 (10.6)
	Confirmed	0 (0.0)	0 (0.0)	0 (0.0)
Treatment outcome	Prevented	9 (10.6)	4 (4.7)	5 (5.9)
	Death	2 (2.4)	2 (2.4)	0 (0.0)
	Unknown	74 (87.0)	74 (87.0)	0 (0.0)
Observation of dog	Observed	1 (1.2)	1 (1.2)	0 (0.0)
	Not observed (< 1 day)	84 (98.8)	79 (92.9)	5 (5.9)

^+^N = 85

*PEP*, Post-exposure prophylaxis

*DBVs*, Dog-bite victims

*Data provided in this table represent a fraction of dog bite victims in Gbarnga city and environs, being individuals who accessed orthodox hospital care by presenting victims for treatment at County-specific reference hospital were enrolled. The normalized dog bite victims calibrated for Gbarnga city and environs is provided in Table 7.

**Table 4:** Profile of dog bite victims and suspected rabies exposed individuals presented for post-exposure treatment at Telewoyan Memorial Hospital, Voinjama city, Liberia, 2008–2013*.

Characteristics	Categories	^+^DBVs (%)	^+^Defaulted in PEP (%)	^+^Completed PEP (%)
Age groups	< 15	40 (54.8)	36 (49.3)	4 (5.5)
	15–30	16 (21.9)	15 (20.5)	1 (1.4)
	31–45	10(13.7)	10 (13.7)	0 (0.0)
	> 45	7 (9.6)	7 (9.4)	0 (0.0)
Gender	Male	42 (57.5)	40 (54.8)	2 (2.7)
	Female	28 (42.5)	28 (38.4)	3 (4.1)
Time care sought	<24 hours	16 (21.9)	15 (20.5)	1 (1.4)
	>24 hours	34 (46.6)	31 (42.5)	3 (4.1)
	Unknown	23 (31.5)	22 (30.1)	1 (1.4)
Anatomical site of bite	Head	13 (17.8)	11 (15.1)	2 (2.7)
	Hands	12 (16.4)	12 (16.4)	0 (0.0)
	Legs	26 (35.6)	23 (31.5)	3 (4.1)
	Trunk	9 (12.4)	9 (12.3)	0 (0.0)
	Multiple sites	13 (17.8)	13 (17.8)	0 (0.0)
Rabies exposure	Not exposed	0 (0.0)	0 (0.0)	0 (0.0)
	Suspected	64 (87.7)	63 (86.3)	1 (1.4)
	Probable	9 (12.3)	5 (6.8)	4 (5.5)
	Confirmed	0 (0.0)	0 (0.0)	0 (0.0)
Treatment outcome	Prevented	5 (6.8)	0 (0.0)	5 (6.8)
	Death	1 (1.4)	1 (1.4)	0 (0.0)
	Unknown	67 (91.8)	67 (91.8)	0 (0.0)
Observation of dog	Observed	1 (1.4)	1 (1.4)	0 (0.0)
	Not observed (< 1 day)	72 (98.6)	67 (91.8)	5 (6.8)

^+^N = 73

*PEP*, Post-exposure prophylaxis

*DBVs*, Dog-bite victims

* Data provided in this table represent a fraction of dog bite victims in Voinjama city and environs, being individuals who accessed orthodox hospital care by presenting victims for treatment at County-specific reference hospital were enrolled. The normalized dog bite victims calibrated for Voinjama city and environs is provided in Table 8.

**Table 5:** Anatomical site of bite injury among dog bite victims* in Buchanan, Gbarnga and Voinjama cities as verified at hospitals (and projected values for 2014–2016 during Ebola virus disease epidemic) in Liberia, 2008–2017.

Study area	Year	Head (%)	Hands (%)	Legs (%)	Trunk (%)	Missing (%)	n (%)
**Buchanan, Grand Bassa County (n = 460)**	2008	5 (35.7)	2 (14.3)	3 (21.4)	4 (28.6)	0 (0)	14 (100)
	2009	2 (6.2)	6 (18.8)	14 (43.8)	10 (31.2)	0 (0)	32 (100)
	2010	24 (12.1)	12 (6.1)	38 (19.2)	35 (17.7)	89 (44.9)	198 (100)
	2011	21 (20.6)	18 (17.6)	37 (36.3)	26 (25.5)	0 (0)	102 (100)
	2012	9 (21.4)	7 (16.7)	18 (42.9)	8 (19.0)	0 (0)	42 (100)
	2013	3 (4.2)	1 (1.4)	6 (8.3)	2 (2.8)	60 (83.3)	72 (100)
	2014	11 (20.8)	8 (15.1)	22 (41.5)	12 (22.6)	0 (0)	53 (100)
	2015	12 (22.2)	8 (14.8)	23 (42.6)	11 (20.4)	0 (0)	54 (100)
	2016	12 (22.6)	8 (15.1)	23 (43.4)	10 (18.9)	0 (0)	53 (100)
	2017	12 (21.8)	9 (16.4)	24 (43.6)	10 (18.2)	0 (0)	55 (100)
	**n**_**2008–2017 (%)**_	**111 (16.4)**	**79 (11.7)**	**208 (30.8)**	**128 (19.0)**	**149 (22.1)**	**675 (100)**
**Gbarnga, Bong County (n = 85)**	2008	1 (12.5)	1 (12.5)	3 (37.5)	3 (37.5)	0 (0)	8 (100)
	2009	2 (33.3)	1 (16.7)	2 (33.3)	1 (16.7)	0 (0)	6 (100)
	2010	2 (20.0)	1 (10.0)	3 (30.0)	4 (40.0)	0 (0)	10 (100)
	2011	3 (16.7)	3 (16.7)	8 (44.4)	4 (22.2)	0 (0)	18 (100)
	2012	9 (27.3)	3 (9.1)	10 (30.3)	11 (33.3)	0 (0)	33 (100)
	2013	2 (20.0)	2 (20.0)	4 (40.0)	2 (20.0)	0 (0)	10 (100)
	2014	6 (25.0)	3 (12.5)	8 (33.3)	7 (29.2)	0 (0)	24 (100)
	2015	7 (26.9)	3 (11.5)	9 (34.6)	7 (26.9)	0 (0)	26 (100)
	2016	7 (24.1)	4 (13.8)	10 (34.5)	8 (27.6)	0 (0)	29 (100)
	2017	8 (25)	4 (12.5)	11 (34.4)	9 (28.1)	0 (0)	32 (100)
	**n**_**2008–2017 (%)**_	**47 (23.9)**	**25 (12.8)**	**68 (34.7)**	**56 (28.6)**	**0 (0)**	**196 (100)**
**Voinjama, Lofa County (n = 73)**	2008	1 (20.0)	1 (20.0)	1 (20.0)	2 (40.0)	0 (0)	5 (100)
	2009	1 (14.3)	1 (14.3)	3 (42.9)	2 (28.5)	0 (0)	7 (100)
	2010	3 (17.6)	3 (17.6)	6 (35.3)	5 (29.5)	0 (0)	17 (100)
	2011	6 (23.1)	3 (11.5)	9 (34.6)	8 (30.8)	0 (0)	26 (100)
	2012	1 (9.1)	2 (18.2)	5 (45.5)	3 (27.2)	0 (0)	11 (100)
	2013	1 (14.3)	2 (28.6)	2 (28.6)	2 (28.6)	0 (0)	7 (100)
	2014	2 (13.3)	3 (20.0)	6 (40.0)	4 (26.7)	0 (0)	15 (100)
	2015	3 (18.8)	3 (18.8)	6 (37.5)	4 (25.0)	0 (0)	16 (100)
	2016	3 (16.7)	3 (16.7)	7 (38.9)	5 (27.7)	0 (0)	18 (100)
	2017	3 (15.8)	4 (21.1)	7 (36.8)	5 (26.3)	0 (0)	19 (100)
	**n**_**2008–2017 (%)**_	**24 (17.0)**	**25 (17.7)**	**52 (36.9)**	**40 (28.4)**	**0 (0)**	**141 (100)**

Projection Equations was used for computation of 2014–2017

**Buchanan, Grand Bassa**

Head (Y_t_ = 9.867+0.229t); Hands (Y_t_ = 7.267+0.114t); Leg (Yt = 16.733+0.743t); Trunk (Y_t_ = 16.667–0.714t)

**Gbarnga, Bong**

Head (Y_t_ = 0.467+0.771t); Hands (Y_t_ = 0.533+0.371t); Leg (Y_t_ = 1.6+0.971t); Trunk (Y_t_ = 1.667+0.714t)

**Voinjama, Lofa**

Head (Y_t_ = 1.867+0.086t); Hands (Y_t_ = 1.2+0.229t); Leg (Y_t_ = 2.933+0.4t); Trunk (Y_t_ = 3.067+0.171t)

*Where multiple bites were sustained by a victim, the victim was assigned to one of the anatomical locations bitten that portends highest probability of developing rabies.

In addition, a descriptive statistical summary of non-hospital treated DBVs was audited and presented in simple tables (Tables 6, 7 and 8), based on data generated from household questionnaire survey of DBVs. The tables summed victims that did and those that did not visit an orthodox health facility in Buchanan, Gbarnga and Voinjama cities, respectively within the 10-year period. Total DBVs presented at the hospitals were estimated based on the ratio of 2017 cases presented at each of the designated county level referral hospital per city, compared to 2017 enhanced reporting from several health facilities in each city/county [14].

**Table 6:** Proportion of hospital presented dog bite victims compared with non-hospital visiting victims used to compute normalized annual cases of dog bite victims in Buchanan City and environs, Grand Bassa County, Liberia, 2008–2017.

Year	Buchanan and Environs				
	*Hospital cases*, x* *(100%)*	*H*_*i1*_ 60%** of x* (a)	*H*_*i2*_ 40%** of x* (b)	Others/ Non-hospital cases [38%*** of (a+b)] = c	Total (*T*_*i*_ *=* a+b+c)
2008	38	25	13	14	52
2009	53	32	21	20	73
2010	312	187	125	119	431
2011	170	102	68	65	235
2012	90	54	36	34	124
2013	100	60	40	38	138
2014	147	88	59	56	203
2015	143	86	57	54	197
2016	150	90	60	57	207
2017	95	57	38	36	131

Key

* Hospital cases calculated based on the ratio of 2008:2017 (or 2009, 2010–2016 respectively) from initial records of single selected county referral hospital per city reporting cases to the MoHSW, compared to 2017 enhanced DBVs reporting health centers to Liberia National Institute of Health following EVD outbreak in the country.

** In Buchanan, out of 10 clinics or human hospitals rather than one (i.e. the Liberian Government Hospital), only 57 DBVs out of 95 DBVs (60% cases) in 2017 were reported to the Liberian Government Hospital.

*** Based on questionnaire survey, 140/365 = 38.35% or 38% of DBVs did not visit any orthodox health care facility.

**Table 7:** Proportion of hospital presented dog bite victims compared with non-hospital visiting dog bite victims used to compute normalized annual cases of dog bite victims in Gbarnga City and environs, Bong County, Liberia, 2008–2017.

Year	Gbarnga and Environs				
	Hospital cases x* *(100%)*	*H*_*i1*_ 4.7%** of *x* (a)	*H*_*i2*_ 95.3%** (b)	Others/ Non-hospital cases [39%*** of (a+b)] = c	Total (*T*_*i*_ *=* a+b+c)
2008	170	8	162	66	236
2009	128	6	122	50	178
2010	213	10	203	83	296
2011	383	18	365	149	532
2012	702	33	669	274	976
2013	213	10	203	83	296
2014	191	9	182	74	265
2015	362	17	345	141	503
2016	404	19	385	158	562
2017	64	3	61	25	89

Key

* Hospital cases calculated based on the ratio of 2008:2017 (or 2009, 2010–2016 respectively) from initial records of single selected county referral hospital per city reporting cases to the MoHSW, compared to 2017 enhanced DBVs reporting health centers to Liberia National Institute of Health following EVD outbreak enhances surveillance in the country.

** In Gbarnga, out of 23 clinics/human hospitals rather than one (Phebe Hospital and Nursing School), only 3 DBVs out of 64 (4.7% cases) in 2017 were from Phebe Hospital and Nursing School.

*** Based on questionnaire survey, 148/377 = 39.2% or 39% of DBVs did not visit any orthodox health care facility in Gbarnga.

**Table 8:** Proportion of hospital presented dog bite victims compared with non-hospital visiting dog bite victims used to compute normalized annual cases of dog bite victims in Voinjama City and environs, Lofa County, Liberia, 2008–2017.

Year	Voinjama and Environs				
	Hospital cases x* *(100%)*	*H*_*i1*_ 23.7%** of *x* (a)	*H*_*i2*_ 76.3%** (b)	Others/ Non-hospital cases [42%*** of (a+b)] = c	Total (*T*_*i*_ *=* a+b+c)
2008	21	5	16	9	30
2009	29	7	22	12	41
2010	71	17	54	30	101
2011	108	26	82	45	153
2012	46	11	35	19	65
2013	29	7	22	12	41
2014	58	14	44	24	82
2015	67	16	51	28	95
2016	75	18	57	32	107
2017	38	9	29	16	54

Key

* Hospital cases calculated based on the ratio of 2008:2017 (or 2009, 2010–2016 respectively) from initial records of single selected county referral hospital per city reporting cases to the MoHSW, compared to 2017 enhanced DBVs reporting health centers to Liberia National Institute of Health following EVD outbreak in the country.

** In 2017, there were 21 clinics or human hospitals, rather than one (Tellewoyon Memorial Hospital) that reported DBVs from Voinjama and environs in Lofa County. Only 9 DBVs out of 38 (23.68% cases) were from Tellewoyon Memorial Hospital in 2017.

*** Based on questionnaire survey, 146/350 = 41.7% or 42% of DBVs did not visit any orthodox health care facility in Voinjama.

#### Spatio-temporal scan statistics

Individual DBV was presented as map point in SaTScan 9.0 environment after they were first converted by site name to map point. A case file of DBVs with time (date) of occurrence in each city was created. Using Kulldorff permutation model [24], a space-time scan was used to analyze and identify the most likely space-time cluster in each city. The Kulldorff’s space time scan statistic was preferred for each city based on its cylindrical window, in which the circular base represents the geographic area of the potential cluster of dog bite and the vertical arm represents the time span. The null hypothesis of space-time permutation scan assumes that the relative risk of occurrence of DBV is the same within the cylindrical window compared with outside it. The model controls for co-variants and eliminates preselection bias by not specifying *a priori* the observed set of cases within a cluster [22, 23].

Spatial size of the scan window was limited to 50% of the total population per city. This was based on reported cases of dog bite from 2008 to 2013 (n = 460, 85 and 73 in Buchanan, Gbarnga and Voinjama, respectively), estimation for 2014–2017 [19], and actual reported cases in 2017. In each analysis, cluster radius of 1.0 km with time scale of one month was specified. Statistical significance of a cluster was determined by comparing the expected against observed number of cases of DBVs based on the null distribution obtained through Monte Carlo simulation with 999 replications and significance level was set as 0.05. The window with the maximum test statistic was considered the cluster least likely to be due to chance (primary cluster). Other windows with statistically significant test results were considered as secondary clusters. Detected clusters were presented in a set of thematic maps of DBVs for Buchanan, Gbarnga and Voinjama using ArcGIS 10.1 environment (Environmental Systems Research Institute, Redlands, CA).

#### Estimation of annual human deaths due to suspected rabies cases, 2008–2017

The probability of developing rabies was calculated based on the model designed by Cleaveland *et al*. [28], and applied by Knobel *et al*. [29] and Tenzin *et al*. [30]. The model comprises a set of ten probability steps that incorporate the distribution of bite injuries on different parts of the body of victims, and the probability of a victim developing rabies, based on verified clinical records. The decision tree for determining the probability of rabies following the bite of a suspect rabid dog has been described by Tenzin et al. [30]. The probability of human deaths resulting from the bite of suspected rabid dog, *PR*, was estimated using the following model equation:

PR=P1x[(P2xP6)+(P3xP7)+(P4xP8)+(P5xP9)]x(1–P10)

where, *P1* is the probability of suspected rabid dog being rabid; *P2* is the probability of bite injury to the head; *P3* is the probability of bite injury to the hands; *P4* is the probability of bite injury to the trunk; *P5* is the probability of bite injury to the legs; *P6* is the probability of developing rabies following bite injury to the head; *P7* is the probability of developing rabies following bite injury to the hands; *P8* is the probability of developing rabies following bite injury to the trunk; *P9* is the probability of developing rabies following bite injury to the legs; and *P10* is the probability of receiving post-exposure treatment if bitten by a suspected rabid dog.

The probability of developing rabies following the bite of a rabid dog to the head (P6), hands (P7), trunk (P8) and legs (P9) were taken to be 45.0%, 28.0%, 5.0% and 5.0%, respectively, as previously reported [28–30].

The total number of suspected human rabies cases per year, N, was estimated by:

N=(ixQxPR)/100,000

where, *i is* the incidence of suspected rabid dog bites per 100,000 per year; *Q is* the population at risk; and *PR* is the probability of a human death following a bite from a suspected rabid dog [28–30].

The population distribution of the three selected cities and environs was calculated based on Liberia 2008 census (which remains the current, official report for the country), and estimated annual growth rate of 1.4% in Buchanan and environs, Grand Bassa County, 1.0% in Gbarnga and environs, and 1.3% in Voinjama and environs in 2008–2011 [13].

## Results

### Categorical description of age, gender and anatomical site of dog bite injury on human victims, 2008–2013

**Fig 3:**
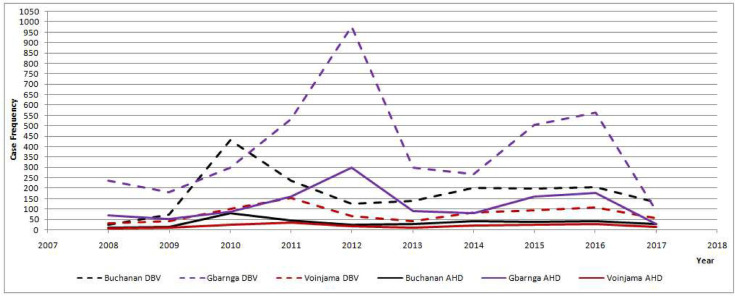
Source: Original data gathered from this study on normalized yearly prevalence of dog bite victims and human deaths due to rabies in three cities and environs, Liberia, 2008–2017.

Of the total of 618 DBVs presented for treatment at the three enlisted reference hospitals in the three selected cities during the first 6-year period, 2008–2013, Buchanan city recorded 460 DBVs, Gbarnga city recorded 85 DBVs and Voinjama city 73 DBVs. Majority (363, 58.74%) of DBVs were male compared to females 255 (40.78, n = 618) (Table 1). Children (age group < 15 years old) accounted for the highest proportion of the DBVs in the three cities (n = 275, 44.5%), followed by youths (age group 15–30 years; n = 152, 24.6%), young adults (age group 31–45 years; n = 102, 16.5%), and adults (>45 years; n = 84, 13.6%), in that order (Table 1 and Fig 3).

The anatomical site of dog bite injury was predominantly on the legs 178 (28.8%, n = 618), followed by head 101 (16.3%, n = 618), multiple sites 97 (15.7, n = 618), and the trunk 40 (6.5%, n = 618). The categorical description of age, gender and anatomical site of bite on human victims in Buchanan, Gbarnga and Voinjama are provided in Tables 2, 3 and 4, respectively, with a summary in Table 5. DBVs below 15 years of age had highest proportion of late presentation among hospital-presented persons, and they had highest number of PEP defaulters in Buchanan (38.2%) and Voinjama (49.3%)). In Gbarnga, age group 15–30 years had highest PEP defaulters (27.1%), compared to persons under 15 years of age (23.5%) (Tables 2, 3 and 4).

### Normalized annual count of dog bite victims, 2008–2017

Normalized annual cases of DBVs, 2008–2017 were computed from the proportion of cases presented at each county designated referral hospital compared to community survey of non-hospital visiting DBVs in each year. Tables 6, 7, 8 and 9 provide city-specific computation of cases of dog bite victims presented at all hospitals and those presented to traditional caregivers. Results revealed that only 57 DBVs out of 95 DBVs in 2017 (that is, 60% of cases), from 10 clinics or hospitals in Buchanan, were reported to the Liberian Government Hospital (Table 7). Thus, a ratio of 60:40 in care-seeking preferences. Household survey indicated that some 140/365 or 38.35% of DBVs in Buchanan did not visit orthodox health care facility. In Gbarnga, normalized hospital cases of DBVs were derived from 23 clinics/hospitals, with only three out of 64 dog bite cases in 2017 (4.7%) obtained from PHNS. Household survey in Gbarnga indicated that, 148/377 or 39.2% of DBVs did not visit any orthodox health care facility (Table 8). Normalized hospital cases of DBVs in Voinjama were derived from 21 clinics. Only nine out of 38 (23.68%) DBVs reported in 2017 were from TMH. Household survey indicated that 146/350 or 41.7% of DBVs did not visit any orthodox health care facility in Voinjama (Table 9).

**Table 9:** Estimation of annual prevalence of human deaths due to rabies in three selected cities and environs, Liberia, 2008–2017.

Year	Buchanan and Environs				Gbarnga and Environs				Voinjama and Environs			
	Total number of suspected rabid dog bite victims (T*i*)	Annual incidence of dog bite / 100,000 population (*i*)	County population at risk (Q)	Annual human death due to suspected rabies (N)	Total number of suspected rabid dog bite victims (T*i*)	Annual incidence of dog bite / 100,000 population (*i*)	County population at risk (Q)	Annual human death due to suspected rabies (N)	Total number of suspected rabid dog bite victims (T*i*)	Annual incidence of dog bite / 100,000 population (*i*)	County population at risk (Q)	Annual human death due to suspected rabies(N)
2008	52	23.13	224,839	4	236	71.75	328,919	21	30	11.11	270,114	2
2009	73	32.02	227,987	6	178	53.58	332,208	16	41	14.98	273,625	3
2010	431	186.44	231,179	34	296	88.22	335,530	27	101	36.44	277,182	8
2011	235	100.25	234,416	19	532	156.99	338,885	48	153	54.49	280,785	12
2012	124	52.12	237,932	10	976	285.15	342,274	88	65	22.85	284,435	5
2013	138	57.14	241,501	11	296	85.62	345,697	27	41	14.23	288,133	3
2014	203	82.90	244,882	16	265	75.90	349,154	24	82	28.09	291,879	7
2015	197	79.34	248310	16	503	142.64	352,646	45	95	32.13	295,673	8
2016	207	82.21	251,786	17	562	157.79	356,172	51	107	35.72	299,517	9
2017	131	51.31	255,311	10	89	24.74	359,734	8	54	17.80	303,411	4
Mean T*i* ± S.D. = 179±106.82				14.3±8.47	Mean T*i* ± S.D. = 393±257.85			35.5±23.25	Mean T*i* ± S.D. = 76.9±38.11			6.1±3.21

### Estimated annual human deaths due to suspected rabies, 2008–2017

An estimated total of 559 DBVs died of suspected rabies in the three cities and their environs during 2008–2017. Majority of DBVs in Buchanan, Gbarnga and Voinjama (47.3%, 58.8% and 46.6%, respectively) that sought medical care were presented more than 24 hours after sustaining dog bite injury (Tables 2, 3 and 4). The estimated annual DBVs for the three cities, from 2008 to 2017, are presented in Table 5. The corresponding parameters for estimating suspected rabies deaths in each of the three cities, based on probability decision tree and anatomical locations of dog-bite injuries are presented in Table 10.

**Table 10:** Predictors of suspected human rabies cases for three selected cities and environs, Liberia, 2012–2017.

Predictor	Description of model variable characteristics	City		
		Buchanan	Gbarnga	Voinjama
P1	Probability of suspected rabid dog being rabid	0.7	0.7	0.7
P2	Probability of bite injury to the head	0.22	0.22	0.16
P3	Probability of bite injury to the hands	0.17	0.14	0.18
P4	Probability of bite injury to the trunk	0.09	0.15	0.13
P5	Probability of bite injury to the legs	0.34	0.36	0.34
P6	Probability of developing rabies following bite injury to the head	0.45	0.45	0.45
P7	Probability of developing rabies following bite injury to the hands	0.28	0.28	0.28
P8	Probability of developing rabies following bite injury to the trunk	0.05	0.05	0.05
P9	Probability of developing rabies following bite injury to the legs	0.05	0.05	0.05
P10	Probability of receiving post-exposure treatment if bitten by a suspected rabid dog	0.03	0.2	0.2
PR	Probability of a human death following a bite from a suspected rabid dog	0.08	0.09	0.08

Annual human deaths estimation parameters were abbreviated as P1, P2, P3, P4, P5, P6, P7, P8, P9, and P10 (Table 10). For Buchanan, these were estimated as 0.7, 0.22, 0.17, 0.09, 0.34, 0.45, 0.28, 0.05, 0.05, and 0.3, respectively. For Gbarnga, they were estimated as 0.7, 0.22, 0.14, 0.15, 0.36, 0.45, 0.28, 0.05, 0.05, and 0.2, respectively, while the estimates for Voinjama were 0.7, 0.16, 0.18, 0.13, 0.34, 0.45, 0.28, 0.05, 0.05, and 0.2, respectively (Tables 6, 7, 8 and 9). Mean yearly cases of DBVs from 2008 to 2017 was 179±106.82, 393±257.85 and 76.9±38.11, respectively in Buchanan, Gbarnga and Voinjama and their respective environs. Annual estimates of the hospital-presented and non-hospital visiting DBVs in Buchanan, Gbarnga and Voinjama, and their respective environs from 2008 until 2017 are shown in Tables 6, 7 and 8, respectively. The estimated Mean Annual Human Deaths due to suspected rabies cases from 2008 to 2017 was 14.3±8.47, 35.5±23.25, and 6.1±3.21 per 100,000 population in Buchanan, Gbarnga and Voinjama cities and environs, respectively (Table 9).

### Space-time clusters of dog bite victims

**Fig 4:**
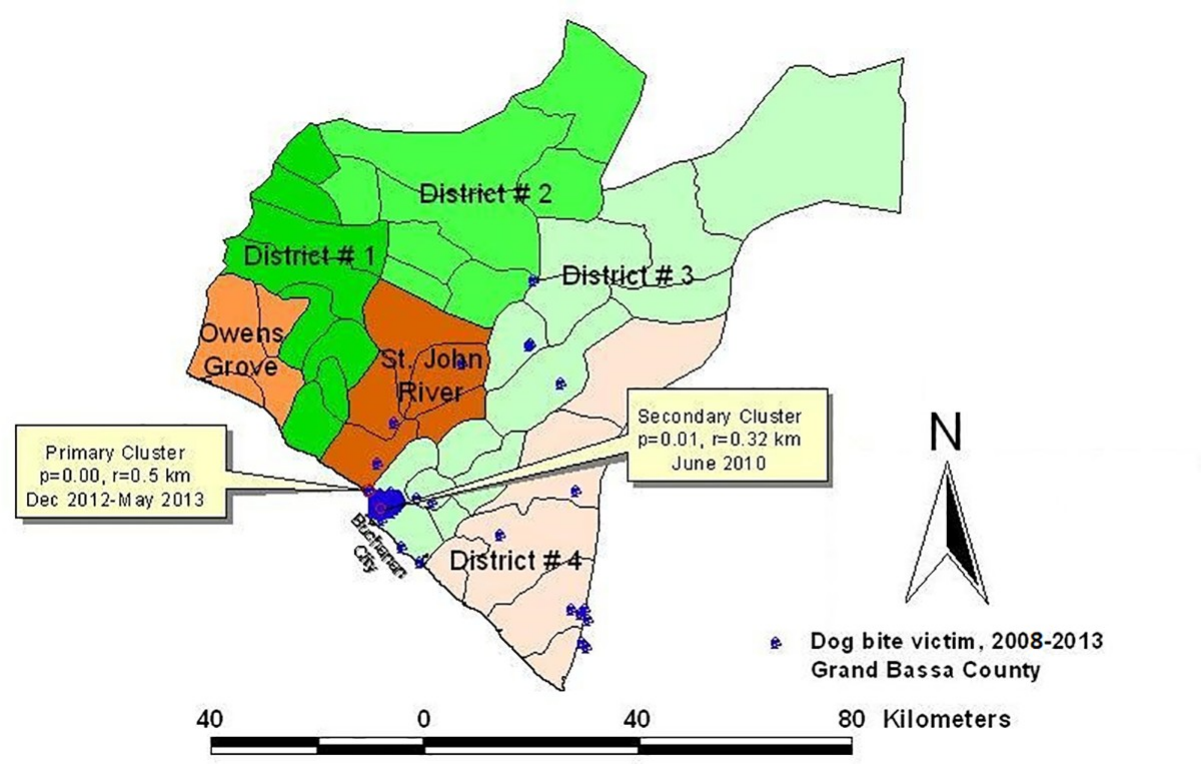
Source: Original map created using ArcGIS 10.1 environment (Environmental Systems Research Institute–ESRI) with datasets from anonymized patient data and space-time cluster analysis results from SatScan 9.0 environment.

**Fig 5:**
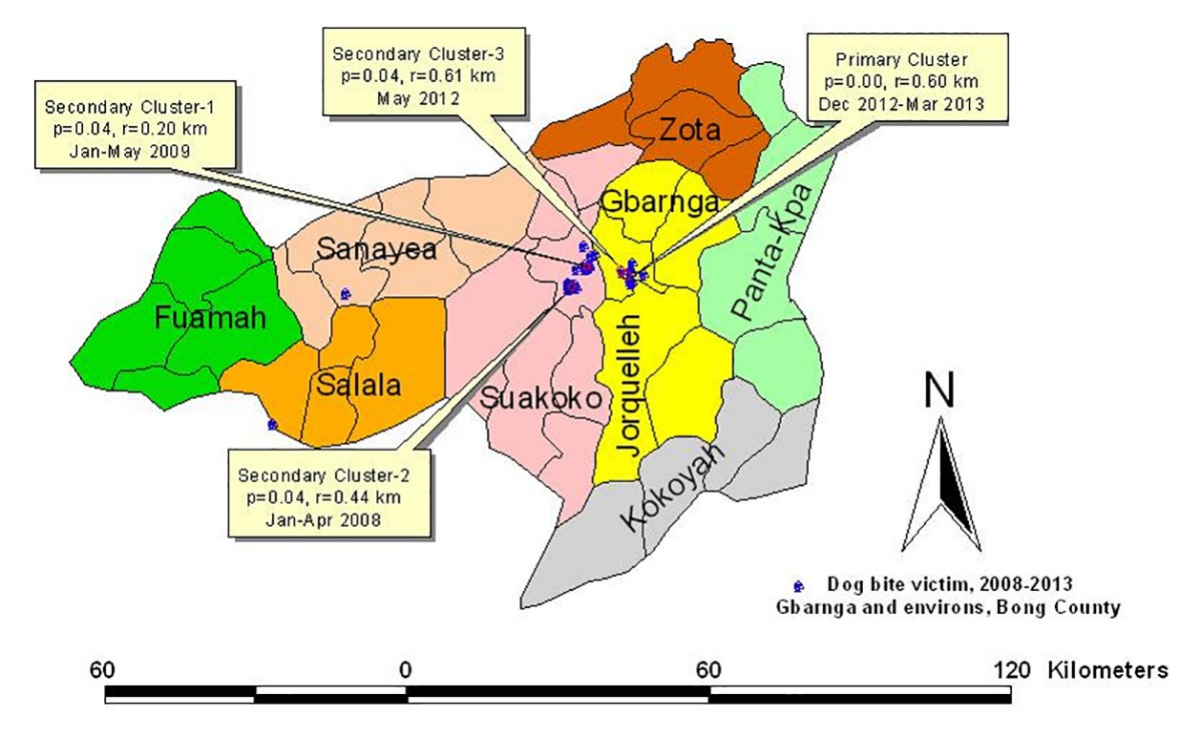
Source: Original map created by the authors using ArcGIS 10.1 environment (Environmental Systems Research Institute–ESRI) with datasets from anonymized patient data and space-time cluster analysis results from SatScan 9.0 environment.

There were two significant space-time clusters in Buchanan. The most significant cluster occurred from May to December 2013. The second cluster occurred earlier, in June 2010, and lasted for only 30 days (*p*<0.013), with a radius of 0.32 km (Table 11; Figs 4 and 5). Four significant space-time clusters of DBVs were detected in Gbarnga City. One of the clusters had a duration of 31 days. In Voinjama, dog bite events were diffuse in space and time without a significant cluster. Notable about these clusters was that they were located within city environment, or in peri-urban areas, relative to other locations outside the clusters. This spatial pattern appear to indicate that persons living closer to county-specific reference hospital were more prone to present victims of dog bite to orthodox hospital nearby compared to people that lived farther away from the hospitals but still within the same County.

**Table 11:** Primary and secondary space-time clusters of dog bite victims presented to County General Hospitals in Buchanan and Gbarnga, Liberia, 2008–2013.

City	Type of cluster	Time frame	Number of cases		Geo-coordinates/ Radius of cluster	Test statistic	P-value
			Observed	Expected			
**Buchanan,** Commonwealth District, Grand Bassa County	Most likely (Principal)	2013/5/1 to 2013/12/31	20	2.95	5.909500° N, 10.062400° W/0.5 Km	21.55	0.00
	Secondary	2010/6/1–2010/6/30	17	4.60	5.881100° N, 10.044700° W/0.32 Km	9.99	0.01
**Gbarnga,** Jorquelleh District, Bong County	Most likely (Principal)	2012/12/1 to 2013/3/31	7	0.94	7.012496° N, 9.480971° W/0.60 Km	8.21	0.00
	Secondary 1	2009/1/1/ to 2009/5/31	3	0.14	7.022058° N, 9.546336° W/0.20 Km	6.36	0.04
	Secondary 2	2008/1/1 to 2008/4/30	3	0.14	6.993919° N, 9.584991° W/0.44 Km	6.36	0.04
	Secondary 3	2012/5/1 to 2012/5/31	3	0.14	7.023661° N, 9.498645° W/0.61 Km	6.36	0.04

## Discussion

The study was conducted to improve dog bite victim survey in three selected cities of Liberia using an explanatory sequential mixed-method study. This included retrospective clinical case review of dog bite victims, household questionnaire survey of victims’ care-seeking preferences and multi-criteria analysis of their post-exposure prophylactic treatment compliance. All these were done to estimate both the absolute number of annual human deaths due to suspected rabies cases and the respective *prevalence* of cases in the three selected cities of Liberia. Thus, we conducted city-specific data audit of dog-bite victims in Buchanan, Gbarnga and Voinjama cities and used a standardized clinical decision tree model, earlier proposed [30] to compute the annual human deaths in each of the three cities. By accounting for previously unreported dog-bite victims and suspected rabies cases, a more accurate estimate of DBVs and suspected rabies cases were determined as the true burdens of rabies in each of Buchanan, Gbarnga and Voinjama cities, along with their respective county environs in Liberia, 2008–2017. We here reported the mean yearly prevalence of dog bite victims as 179±106.82, 393±257.85 and 76.9±38.11, respectively; while estimated mean annual human deaths attributed to suspected rabies cases were 14.3±8.47, 35.5±23.25, and 6.1±3.21 per 100,000 population in Buchanan, Gbarnga, and Voinjama cities, respectively with their environs,. In all, a total of 559 humans were estimated to have died of suspected rabies cases in the three cities over the 10-year period, 2008–2017.

The one health audit of dog bite victims’ data in the three cities was made possible through collaboration of the Ministry of Health and Social Welfare (MoHSW), with the Ministry of Agriculture (MoA), Cuttington University, Liberia and the University of Ibadan, Nigeria. Thus, the two critical government agencies that were needed to enlist their employees who were willing, able, and available for participation in human-animal disease survey, laboratory detection and reporting in Liberia supported the project. Two personnel, one each from MoHSW and MoA enrolled with the CCPZ to embark on a postgraduate program in human-animal disease surveillance at the University of Ibadan, Nigeria. The two sectors thereby worked with the academia to pool together essential data from animal and human health sectors. Questionnaire survey were jointly designed and conducted to gather additional field and archival specimens of suspected dog and human rabies cases for confirmation of rabies at the grassroots level in the three Liberian cities from 2012 to 2017. All secondary data used in this study were provided by the Liberia Ministry of Health and Social Welfare. All the data were shared with personnel of the Central Agricultural Research Institute (CARI) and academia in one health partnership.

Gbarnga city had the highest (approximately 36 deaths per 100,000 population) of annual human deaths due to suspected rabies cases out of the three cities. The 36 cases were much lower than what was reported [2] for Monrovia, the capital city of Liberia, being 155 AHD due to rabies, about the same period. The Gbarnga rabies burden was comparable to reported estimate for Plateau State, Nigeria, and Abidjan in Cote d’Ivoire, being an average annual human deaths of 40 per 100,000 population, due to rabies [1, 31, 32]. Gbarnga contrast with Monrovia is to be expected, since there is a high human population density difference between the two Liberian cities, with Monrovia, having about ten times the population density of Gbarnga [12, 13]. Yet, the situation in Gbarnga portends a relatively high prevalence of suspected rabies deaths when compared to Plateau State of Nigeria. In each of the 10-year chronological prevalence, the annual human deaths due to suspected rabies cases in Buchanan was higher than Voinjama city, but comparable to other municipalities in West Africa, including Techiman Municipality in the Middle Belt of Ghana [33].

The two space-time clusters (Table 11) of dog bite victims identified in Buchanan and the four in Gbarnga city (based on hospital-presented cases only) were proximal to the locations of each reporting hospital facility during 2008–2013, whereas rabies-susceptible dogs were present across the entire city and county landscapes as reported by Jomah et al. [34] based on rabies neutralizing antibody survey, 2015. This finding corroborates the tendency of dog bite victims to self-medicate or present to traditional caregivers in rural areas than to conventional hospitals among low income populace in Liberia [2]. A hospital-based surveillance system would usually miss this segment of the public, except where additional survey is done to capture non-hospital visiting sub-group. Poverty and related low- or non-formal education have been associated with non-hospital care-seeking tendencies among people in Liberia [8, 9] in general and dog bite victims in particular [2]. Often the victims were unable to afford the cost of rabies vaccines for post-exposure prophylaxis, and would naturally seek care from traditional caregivers. This is similar to the findings in Ilorin, a city of Nigeria with notable traditional sector [35], as well as in the slum areas of Monrovia in Liberia [2, 36], and slums of Abidjan in Cote d’Ivoire [32].

The detection of cluster radii lower than 1.0 km and time scale larger than a month, which were different from those earlier specified at statistical analysis were due to *a priori* guide to be analysis. It was notable that shortly after Ebola virus disease epidemic ended in 2017, the number of hospitals included in the integrated disease surveillance system had remarkably increased from three hospitals only (one each from the three selected counties, of Grand Bassa, Bong and Lofa, respectively) as obtained during 2008 to 2014, to a minimum of ten hospitals/clinics per county in each of the 15 counties of Liberia by 2017 [14]. This transformation was facilitation by the WHO following Ebola virus disease outbreak that attracted global attention and support to Liberia [14, 20, 21].

We have exercised high caution in our claims about preliminary laboratory confirmation backing of the secondary data sourced form hospital records and the MoHSW data because of the retrospective nature, 2008–2017 that did not have accompanying laboratory confirmation of each of the rabies suspected cases. However, rabies suspect status and the deterministic model of dog bite victims here derived conformed to WHO 2004 case categorization. We have reported that one of the RABV strains (MF765758) isolated in Liberia clustered with China Lineage 2 of dogs. This strain co-circulated with two other strains of Africa lineages [6]. Five other RABV strains, two each first isolated in France (GU992321, GQ918139), and India (KF535200, KF535201) and one from China (DQ875050) have since been added by Zaho et al. [37], to the list of RABV strains that were 99% similar to Liberia MF 765758 strain [6], thus, being phylogenetically of the same lineage, China Lineage 2 (cosmopolitan). These indices have added value to closing the critical data gap in the 10-year prevalence of suspected rabies cases [38] in the three cities. The sequential mixed-method approach here used could be adaptable to other counties of Liberia and West African countries [10, 11, 25, 38–40].

These authors have learned that a horizontal [41] and a vertical line of interaction with stakeholders in rabies surveillance are essential to achieve the one health action in Liberia here reported. The achievement would be sustained through the same lines of engagement and research coordination. As a result, a forum for Rabies in West Africa (RIWA), which was inaugurated in Ibadan, Nigeria in December 2012 [42] had administratively incorporated a board of trustees for a Society for Rabies in West Africa in November 2017. The new incorporation will enhance a sustainable coordination of the improvement now achieved in rabies surveillance, by further facilitating partnership network with the Central Veterinary Laboratory in Liberia and with regional and global veterinary laboratory diagnosticians, such as from the University of Ibadan CCPZ, the Global Alliance for Rabies Control, the OIE-FAO-WHO tripartite and the Swiss Tropical and Public Health Institute. In addition, fostering partnership with the World Association of Veterinary Laboratory Diagnosticians, would enhance quality in veterinary diagnostic services as required by OIE [43], and supported by the Pan-African Association of Veterinary Laboratory Diagnosticians.

The present study presented a 10-year chronological estimates of annual prevalence of human deaths due to suspected rabies cases in Buchanan, Gbarnga and Voinjama cities of Liberia with their local environs, 2008–2017. This information will be useful for planning more effective control and stepwise actions to achieving the goal that no human should die of rabies by 2030.

## Supporting information

S1 PRISMA checklist STROBE Statement—Checklist of items that should be included in reports of cross-sectional studies. (DOC)
